# Orthognathic Surgery with Interdisciplinary Digital Planning in Patients with Geroderma Osteodysplasticum: A Case Report

**DOI:** 10.3390/jpm13111578

**Published:** 2023-11-04

**Authors:** Konrad Tolksdorf, Stefan Schultze-Mosgau, Collin Jacobs, Stephanie Tietz, Christoph-Ludwig Hennig

**Affiliations:** 1 Department of Oral and Maxillofacial Surgery/Plastic Surgery, University Hospital Jena, Am Klinikum 1, 07747 Jena, Germany; 2 Department of Orthodontics, Center of Dental Medicine, Jena University Hospital, An der Alten Post 4, 07743 Jena, Germany

**Keywords:** geroderma osteodysplasticum, dentofacial deformities, orthognathic surgery

## Abstract

Patients with geroderma osteodysplasticum (GO) often times have dentofacial deformities and benefit from orthognathic surgery. Because of generalized osteopenia, operations must be prepared even more meticulously than usual, and the higher risk of unfortunate fractures (bad splits) should be explained to the patients in detail. This case report is intended to portray a digital, interdisciplinary and patient-individualized planning of orthognathic surgery. It points out the individual steps that must be considered and how they can be advantageously used in patients with underlying diseases or syndromes such as GO. Through a careful digital representation of the surgical options, production of the digitally modeled splints, 3D printing and good manual surgical implementation, the quality of life of patients with GO can be increased through orthognathic surgery. Both the functions in the oral, maxillofacial region and the patient’s appearance in the case presented here benefited from the interdisciplinary, individualized and digital treatment approach.

## 1. Introduction

Geroderma osteodysplasticum (GO) was first described in 1950 in a Swiss family and initially interpreted as a form of progeria [1]. It is an autosomal recessive hereditary disease [2,3], with a loss-of-function mutation in SCYL1BP1 (alias: GORAB, RAB6-Interacting Golgin) on chromosome 1q24.2 [4,5,6]. This mutation leads to alterations of the connective tissue, which generates a typical clinical picture. Previously disparagingly called “Walt-Disney-Dwarfism”, patients with GO are characterized by prematurely aged features and a relatively short stature [1]. Archetypal characteristics are lax and wrinkly skin, joint hyperflexibility, mandibuläre prognathia and an underdevelopment of the malar prominence. The osteoporosis-like decalcification of the bones often leads to vertebral compression fractures and an abnormally increased tendency for fractures [3,7,8,9]. Frequently, patients show crowded teeth and a thin alveolar bone [10]. Apical hypercementosis causes a radiolucent halo around the roots, and the previously mentioned hypoplastic mandibular bone leads to a superimposition of the mandibular canal [11]. There are differential diagnoses that have a similar clinical picture. These include syndromes such as cutis-laxa syndrome, wrinkly skin syndrome, De-Barsy syndrome and Ehlers–Danlos syndrome. GO is relatively rare, with a prevalence of 1:1,000,000; therefore, little is known about its treatment and progression. Treatment of the cause is not possible, but symptoms such as prevailing osteoporosis are predominantly reported, which is treated with bisphosphonates. An interdisciplinary exchange is important to achieve a good treatment outcome for the patients, as many departments are involved due to the complex clinical picture. This case report describes the individualized, patient-specific and interdisciplinary approach to the combined orthodontic and maxillofacial treatment and digital surgical planning of jaw deformities in GO. The advantages and disadvantages of personalized, interdisciplinary digital surgical planning will be presented.

## 2. Case Report

A 31-year-old female patient presented to the department of oral- and cranio-maxillofacial surgery/plastic surgery of the Jena University Clinic. She was dissatisfied with her facial and dental features and reported a year-long odyssey through several dental and orthodontic offices. Diagnosed with GO and being in a post multiple vertebral fracture status, no dentist saw a way to finally help her.

**Figure 1:**
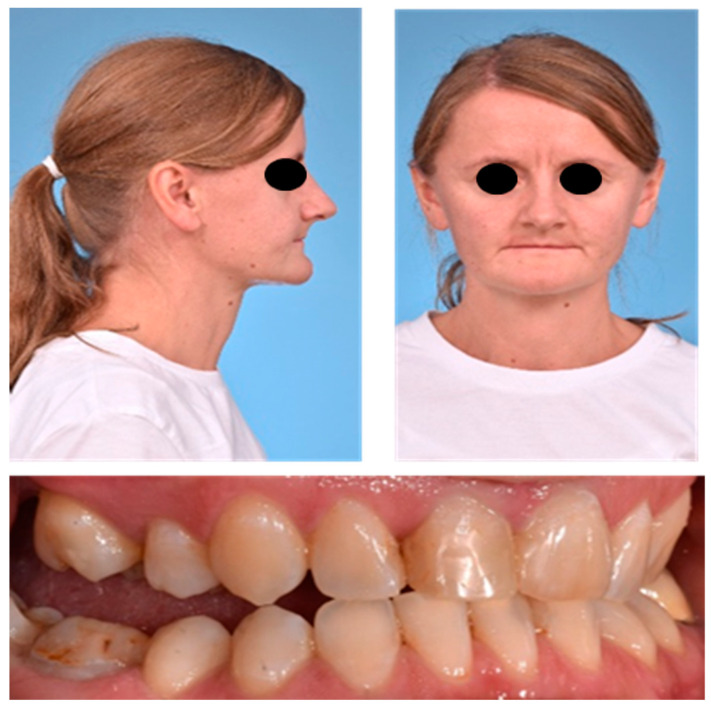
The patient’s facial features before any surgical intervention: typical lax, wrinkly skin and droopy cheeks with an altogether older appearance than one would assume regarding the chronological age. The patient had a bilateral open bite.

In our clinical examination, the 1.6 m tall patient presented with typical lax and wrinkly skin especially around the hands, feet and abdomen as well as droopy cheeks, resulting in an altogether older appearance than one would assume regarding chronological age (Figure 1).

The previous treating dentists had extracted multiple maxillary teeth to reduce maxillary crowding caused by a hypoplastic maxilla. Nevertheless, they could not achieve normal occlusion. The orthodontic findings showed a dental class III occlusion with a lateral open bite on the right and left. The patient had a pronounced Curve of Spee and a head bite situation in the anterior region.

**Figure 2:**
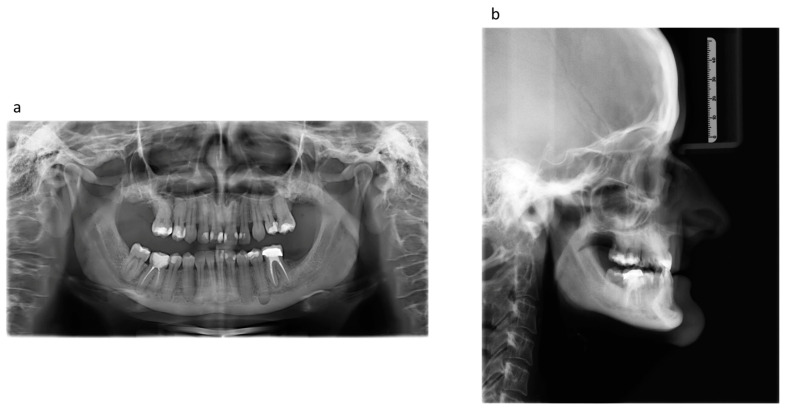
(**a**) Orthopantomography before any orthodontic and surgical intervention: note the typical hypercementosis with apical radiopaque halos and the dental roots superimposing the mandibular canal. (**b**) The lateral cephalogram before any orthodontic and surgical intervention: skeletal class III according to ANB (−2.2°) and WITS (−6.6 mm) with an orthognathic maxilla and a manifest prognathous mandible.

In the radiographic examination, maxillary retro- and mandibular prognathia was apparent. In the lateral cephalogram, the patient displayed a skeletal class III according to ANB (−2.2°) and WITS (−6.6 mm) with an orthognathic maxilla and a manifest prognathous mandible. The patient had a horizontal facial skull structure (ML-NL 11.9°). The patient’s hypercementosis generated apical radiopaque halos, and the dental roots superimposed the mandibular canal (Figure 2). 

**Figure 3:**
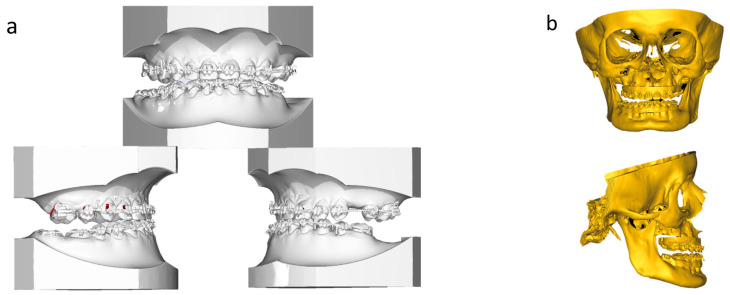

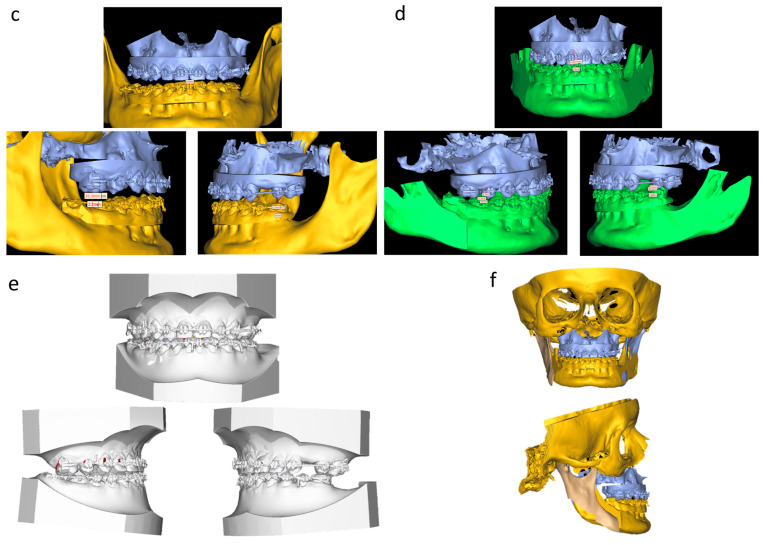
Digital planning was conducted with the software Materialise Mimics and Proplan CMF (Materialise NV, Leuven, Belgium) and the software OnyxCeph (Images Instruments, Chemnitz, Germany): (**a**) digital models before surgery, (**b**) skeletal situation before surgery, (**c**) digital planning Le-Fort-I-osteotomy operation maxilla, (**d**) digital planning BSSO operation mandibular, (**e**) digital model planning after surgery and (**f**) digital skeletal situation planning after surgery.

After performing surgical-assisted palatal expansion, extensive diagnostics, and patient briefing and consent, we performed double jaw surgery with a relocation of the maxilla and rotation of the mandibula (Le-Fort-I-osteotomy, BSSO). The operation was planned in an interdisciplinary digital way. Digital planning was conducted with the software Materialise Mimics and Proplan CMF (both Materialise NV, Leuven, Belgium) and the software OnyxCeph 3.2 (Images Instruments, Chemnitz, Germany) (Figure 3).

**Figure 4:**
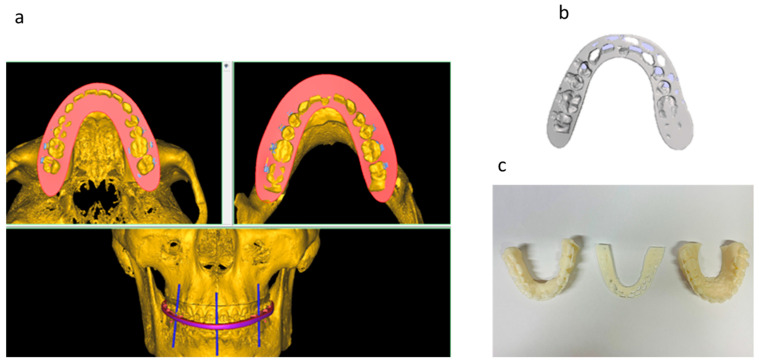
Digital splint planning: (**a**) splint planning in Proplan CMF (Materialise NV, Leuven, Belgium), (**b**) the digital target splint and (**c**) the 3D-printed target splint and models with Asiga-Max 3D printer (Scheu-Dental GmbH, Iserlohn, Germany).

In the process, displacement distances for the maxilla and mandible were determined, and the surgical splints were printed with the Asiga-Max 3D printer (Scheu-Dental GmbH, Iserlohn, Germany) (Figure 4).

**Figure 5:**
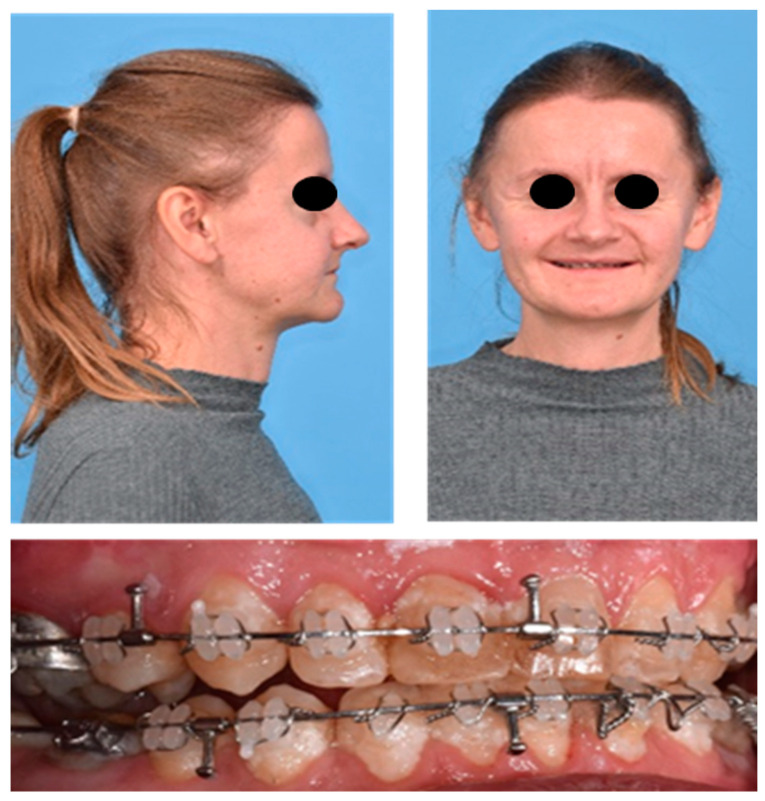
The patient’s facial features after the orthognathic surgery: after the surgery, neither mandibular prognathia nor open bite were present anymore.

**Figure 6:**
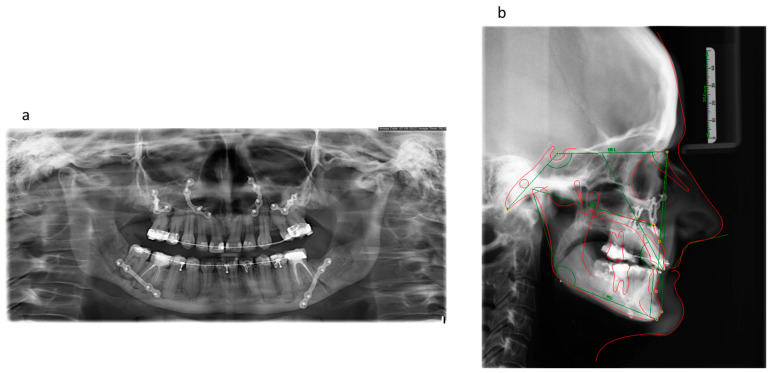
(**a**) Orthopantomography with orthodontic and surgical intervention and (**b**) the lateral cephalogram with orthodontic treatment and after surgical intervention: skeletal class I according to ANB (3.4°).

Even though we executed the osteotomy extremely carefully, an unintentional fracture in the lingual area of the ascending ramus appeared. Fortunately, this fracture had no influence on the success of the osteosynthesis, and we obtained a satisfying surgical result. After some pondering of refixation, the fractured part was ultimately removed. The patient had no increased pain and was satisfied with the result. Chewing, biting and the patient’s esthetic appearance significantly improved. No change was observed in speech and swallowing. After a regular postoperative healing period, the surgical outcome was determined with a lateral cephalogram. Postoperatively, skeletal class I according to ANB (3.4°) was present (with a WITS -3 mm and ML-NL 5.5°) (Figure 5 and Figure 6).

## 3. Discussion

The genetic and phenotypic alterations in patients with GO are frequently described in the literature [6,12,13,14]. These changes and the degree of expression seem to be very different. Often the change, the premature aging of the skin and the disturbance of the connective tissue development are described. Less is known about the dental and maxillofacial treatment of this disease profile. Due to the different degrees of manifestation, the therapy should be personalized and adapted to the patient [9,15,16]. The facial skull and the jaws are also affected and often altered in the context of skeletal deformities. These often affect the function of speech, articulation and myofunctional processes such as swallowing and eating. Furthermore, the external appearance may be altered. In addition to wrinkly skin, patients with GO look older because they have abnormal vertical and sagittal craniofacial configurations, and the chin is very prominent. Thus, orthodontic and maxillofacial surgery treatment can have a positive influence on function and esthetics. For planning the procedure and in addition to the expertise of the practitioners, various diagnostic devices such as intraoral scan, x-ray diagnostics, digital photography, face scans as well as a 3D printer are beneficial. There are currently no recommendations for diagnostic planning equipment and software. This means that each practitioner must learn and implement the digital workflow by themselves. At present, many analog techniques are still used for treatment planning and often only include the patient’s occlusion. However, digital planning can be very helpful in patients with certain diseases or syndromes for its possibility to include all the osseous features of the patient. The authors of this report could only find two cases of surgical correction (Le-Fort-I-osteotomy and Le-Fort-I-Osteotomy combined with a bilateral vertical osteotomy of the mandible) of facial malformations in patients with GO, which were published in one article [11]. Here Lustmann et al. first described reduced bone distal to the mandibular lingula. There was no report of any bilateral sagittal split osteotomy of the mandible.

It is important to report on the successful orthognathic treatment of patients with GO, because the positive change in the face and the masticatory system can significantly improve their quality of life. Digitally planned orthognathic surgery is becoming state of the art and can be used in a highly individualized manner [17,18,19]. In the current literature, there is no case describing orthodontic and oral and maxillofacial surgery rehabilitation and the implementation of orthognathic surgery in patients with GO. Likewise, there is little experience with digital surgical planning in this area. Digital diagnosis and treatment planning can be beneficial to achieve a good, individualized treatment outcome. The case report presented here is the first to describe such treatment. Various digital diagnostic devices such as intraoral scanners, face scanners, 3D cameras, 3D printers and 3D radiography such as computer tomography or cone beam computer tomography are used for planning. The digital diagnostic 3D data are then further processed with various software programs and the surgical procedure is planned [20,21,22]. The splints are also digitally designed and then manufactured using CAD/CAM, or printed using 3D printers, and are used for orientation during surgery [23,24,25]. At the moment, there are no recommendations as to which diagnostic devices and software programs can be used. The digital planning and surgical implementation in the patient with GO presented here demonstrates successful treatment results. Since there are currently no comparable case reports, no statement can be made as to how the orthodontic and maxillofacial surgical treatment of patients with GO could have proceeded as an alternative to the procedures presented here. Nevertheless, analogous planning could have been equally possible. Analog planning would have taken place with impressions of the upper and lower jaw with facebow and articulation in an articulator to represent the pre-surgical situation. Then, based on the radiographs, photos and models, an analog model surgery would have taken place in the articulator. Displacement distances would have been measured by hand, and splints would have been manually constructed by a dental technician on stone models. Good treatment results are possible with both variants, analog or digital planning. A positive aspect of digital planning is the fact that various surgical treatment options can be displayed with good dimensional accuracy and be compared with each other while considering the entire skull. By simulating different treatment options, the most suitable one can be selected. It should be noted, however, that this cannot ensure the success of the operation. Implementation on the patient still depends, to some extent, on the surgeon’s skill.

Evidently, digital planning seems to have advantages, especially for rarely occurring diseases and rarely described surgical procedures. It should also be mentioned, however, that digital preparation still requires more planning time and higher costs than analog procedures.

## 4. Conclusions

This report described how digital orthodontic and maxillofacial planning, together with the implementation of orthognathic surgery, can be performed in patients with GO. Because of the generalized decalcification of the bones, the risk for unfavorable and unanticipated intraoperative fractures is increased. In the future, further exchange on the treatment of patients with GO is desirable. The establishment of a guideline is also conceivable.
